# Utilisation of supplemented l-carnitine for fuel efficiency, as an antioxidant, and for muscle recovery in Labrador retrievers

**DOI:** 10.1017/jns.2017.4

**Published:** 2017-04-03

**Authors:** Jessica L. Varney, J. W. Fowler, W. C. Gilbert, C. N. Coon

**Affiliations:** 1 Four Rivers Kennel LLC, Walker, MO 64790, USA; 2 Threshold Enterprises, Scotts Valley, CA 95066, USA

**Keywords:** l-Carnitine, Canine performance, Dog nutrition, Muscle recovery, Antioxidants, Fuel efficiency, Labrador retrievers, APKm, activity points per km, CPK, creatine phosphokinase, LM, lean mass, ME, metabolisable energy, TAC, total antioxidant capacity, TBARS, thiobarbituric acid reactive substances

## Abstract

The primary goal was to investigate the effects of l-carnitine on fuel efficiency, as an antioxidant, and for muscle recovery in Labrador retrievers. Dogs were split into two groups, with one group being supplemented with 250 mg/d of Carniking™ l-carnitine powder. Two experiments (Expt 1 and Expt 2) were performed over a 2-year period which included running programmes, activity monitoring, body composition scans and evaluation of recovery using biomarkers. Each experiment differed slightly in dog number and design: fifty-six *v*. forty dogs; one endurance and two sprint runs per week *v*. two endurance runs; and differing blood collection time points. All dogs were fed a low-carnitine diet in which a fixed amount was offered based on maintaining the minimum starting weight. Results from Expt 1 found that the carnitine dogs produced approximately 4000 more activity points per km compared with the control group during sprint (*P* = 0·052) and endurance runs (*P* = 0·0001). Male carnitine dogs produced half the creatine phosphokinase (CPK) following exercise compared with male control dogs (*P* = 0·05). Carnitine dogs had lower myoglobin at 6·69 ng/ml following intensive exercise compared with controls at 24·02 ng/ml (*P* = 0·0295). Total antioxidant capacity (TAC) and thiobarbituric acid reactive substance (TBARS) results were not considered significant. In Expt 2, body composition scans indicated that the carnitine group gained more total tissue mass while controls lost tissue mass (*P* = 0·0006) and also gained lean mass while the control group lost lean mass (*P* < 0·0001). Carnitine dogs had lower CPK secretion at 23·06 *v*. control at 28·37 mU/ml 24 h after post-run (*P* = 0·003). Myoglobin levels were lower in carnitine *v*. control dogs both 1 h post-run (*P* = 0·0157; 23·83 *v*. 37·91 ng/ml) and 24 h post-run (*P* = 0·0189; 6·25 *v*.13·5 ng/ml). TAC indicated more antioxidant activity in carnitine dogs at 0·16 mm *v*. control at 0·13 mm (*P* = 0·0496). TBARS were also significantly lower in carnitine dogs both pre-run (*P* = 0·0013; 15·36 *v*. 23·42 µm) and 1 h post-run (*P* = 0·056; 16·45 *v*. 20·65 µm). Supplementing l-carnitine in the form of Carniking™ had positive benefits in Labrador retrievers for activity intensity, body composition, muscle recovery and oxidative capacity.

l-Carnitine is a conditionally essential nutrient that has been shown to have many uses and benefits for the overall health in people and animals. l-Carnitine is essential for transporting long-chain fatty acids into the mitochondria^(^^1^^)^ and has been shown to be necessary for normal cardiac and skeletal muscle function^(^^2^^)^. Although l-carnitine is able to be synthesised in the hepatic and renal systems of humans and dogs^(^^3^^)^, this is not possible within cardiac or skeletal muscle and thus l-carnitine is either absorbed from the diet or biosynthesised by other tissues^(^^4^^)^. l-Carnitine supplementation has gained popularity in both human athletic performance and the companion animal industry in recent years, although few studies have been performed on performance and recovery in canines. It has been suggested that although the vitamins and minerals in commercial diets should be sufficient for less active dogs, they may need to be altered in active canines^(^^5^^)^. In a canine study using greyhound dogs, liquid l-carnitine supplementation induced reduced plasma lactate concentrations and reduced exercise-induced muscle damage during sprinting exercise^(^^6^^)^. Greyhounds, however, primarily have fast-twitch muscle fibres while other breeds have primarily slow-twitch muscle fibres, and so the effect of supplemented l-carnitine may vary between breeds^(^^5^^)^.

Most l-carnitine studies have been performed using human subjects, particularly human athletes, although results have been somewhat conflicting. A double-blind placebo-controlled human study performed on elite athletes found beneficial effects during chronic l-carnitine supplementation on lipid metabolism, evoked muscular potential, VO_2max_, behaviour and biological output. Beneficial effects on physical output, lipid metabolism, muscular function, post-exercise lactate, and urine mucoproteins were also found during acute supplementation^(^^7^^)^. A single-blind study using six human subjects found that l-carnitine supplementation reduced pain and delayed the onset of muscle soreness following eccentric exercise, based on creatine phosphokinase (CPK) assay results and subjective muscle soreness grading^(^^8^^)^. Alternatively, a study comparing l-carnitine's effects on trained human swimmers found no difference on swimming time, swim velocity or post-exercise lactate^(^^9^^)^. Broad *et al.*^(^^10^^)^ found no benefit on human cycling performance but rather that l-carnitine supplementation tended to reduce mobilisation and/or oxidation of fatty acids. The present study was developed based on the promising work performed in human subjects and the need for continued work in the canine-specific response to l-carnitine in performance and recovery aspects.

During the course of two experiments, l-carnitine's effects on food intake, body weight, body composition and activity output during exercise were evaluated. Recovery aspects such as heart rate, body temperature and blood biomarkers before and after were examined. CPK and myoglobin were selected as biomarkers of muscle damage based on the secretion of these enzymes after strenuous exercise^(^^11^^)^. Total antioxidant capacity (TAC) and thiobarbituric acid reactive substances (TBARS) were selected as biomarkers of oxidative stress^(^^11^^,^^12^^)^. Based on previous studies, it was hypothesised that l-carnitine could have beneficial performance and recovery effects for Labrador retrievers during and following a strenuous exercise regimen.

## Materials and methods

All procedures were reviewed and approved by the Institutional Animal Care and Use Committee at Four Rivers Kennel, LLC under protocol FRK-04.

### Animals and housing

A group of forty Labrador retrievers (twenty male/twenty female) ranging from 1 to 4 years of age were utilised in experiment 1. A group of fifty-six Labrador retrievers (twenty-eight male/twenty-eight female) ranging from 1 to 4 years of age were utilised in experiment 2. Sex and colour by group for both experiments are displayed in Table 1. All dogs were allowed free access to outside airing yards for 4 to 6 h daily, weather permitting, and were housed in individual kennels overnight. All dogs had unrestricted access to automatic waterers. Dogs were fed once daily in the morning as per their treatment requirements.

**Table 1.** Demographics of dogs used in experiments 1 and 2 (*n*)

Treatment group	All dogs	Intact males	Altered males	Intact females	Altered females	Black	Yellow	Chocolate
Experiment 1								
Carnitine	20	9	1	10	0	10	9	1
Control	20	9	1	10	0	14	6	0
Experiment 2								
Carnitine	28	13	1	14	0	14	14	0
Control	28	13	1	14	0	14	14	0

### Diets

A low-l-carnitine basal diet was formulated for all dogs in both experiments formulated by Dr George Collings (Collings Nutrition Solutions) and prepared with extrusion equipment at Kansas State University (Manhattan, KS, USA) (Table 2). The same diet formulation from different manufacturing dates was fed during both experiments and the nutrient content of each diet was determined before the start of each study. The quantity of food provided to each dog on a daily basis was set and adjusted based on maintaining a minimum initial starting body weight throughout the study. Feed consumption was determined daily by weighing feed provided and feed refusals.

**Table 2.** Ingredient composition and analysed nutrient content of the low-l-carnitine basal diet

	Ingredient (%)			Nutrient content (%, as-fed basis)	
Ingredient	Expt 1	Expt 2	Nutrient	Expt 1	Expt 2
Maize, ground	42·8550	42·8550	DM	93·6	93·4
Chicken meal	29·0000	29·0000	Moisture	8·00	8·00
Wheat, ground	12·8000	12·8000	Crude protein	25·6	27·4
Rice, brewer's	5·5000	5·5000	Crude fat	14·07	12·8
Beet pulp	5·5000	5·5000	Crude fibre	2·57	2·57
Egg, dried	1·1100	1·1100	Ca	1·26	1·26
Flaxseed	1·1100	1·1100	P	0·89	0·89
Salt, plain	0·5900	0·5900	Ash	6·04	6·04
Potassium chloride	0·5500	0·5500	Methionine	0·54	0·54
Mixed tocopherols	0·2200	0·2200	Lysine	0·98	0·98
l-Lysine	0·2180	0·2180	Na	0·33	0·33
dl-Methionine	0·1920	0·1920	K	0·64	0·64
2011-No K CNS vitamin premix	0·1330	0·1330	Mg	0·12	0·12
2011-01 CNS mineral premix	0·1110	0·1110	Fe	263·45	263·45
Choline chloride 60 %	0·1110	0·1110	Cu (ppm)	20·94	20·94
l-Carnitine	14·9	19·3	Zn (ppm)	233·44	233·44
Metabolisable energy			Linoleic acid	3·82	3·82
kcal/kg	3987	3988			
kJ/kg	16682	16686			
Digestible energy			*n-*6 Fatty acids	3·51	3·51
kcal/kg	4163	4224			
kJ/kg	17418	17673			
Gross energy					
kcal/kg	5033	4620			
kJ/kg	21058	19330			

ppm, Parts per million.

For each experiment, the metabolisable energy (ME) for the low-l-carnitine basal diet was determined using the indicator method^(^^13^^)^. Diet samples and faecal samples from six dogs were collected after each experiment and analysed for crude protein and gross energy using bomb calorimetry (University of Arkansas Central Analytical Laboratory, Fayetteville, AR, USA). l-Carnitine levels in test foods for both experiments were tested using a radioisotopic enzymatic method (Metabolic Analysis Labs, Madison, WI, USA)^(^^14^^)^. The basal l-carnitine levels were slightly higher at 19·3 % (0·048 mg/kcal ME; 0·011 mg/kJ ME) in the diet fed for experiment 2 compared with 14·9 % (0·037 mg/kcal ME; 0·009 mg/kJ ME) in the diet fed for experiment 1 The gross energy (kJ/g) was slightly higher in experiment 1 compared with experiment 2 but the ME was approximately the same (Table 2).

### Added supplements

For both experiments, each dog in the carnitine group was supplemented each day with 3·75 g sugar and 250 mg Carniking™ brand (l-carnitine powder provided by Lonza Ltd (‘Lonza’)), based on manufacturer recommendations. l-Carnitine supplement absorption is primarily passive in mammals and bioavailability of the dose is typically 14–18 %^(^^15^^)^. The control group was supplemented with 4 g sugar. All dogs received the same amount of supplements regardless of weight, although the total intake of l-carnitine was dependent upon food intake (Table 3). Supplements were added to 200 g of food for each dog and fed first, to ensure all dogs were consuming the full amount of l-carnitine and sugar. After the dog had consumed the first 200 g and supplements, they were then given the rest of their meal.

**Table 3.** l-Carnitine (LC) intake per kg body weight (BW) (Mean values)

	Carnitine			Control		
	BW (kg)	Total LC intake (mg)	Total LC (mg/kg BW)	BW (kg)	Total LC intake (mg)	Total LC (mg/kg BW)
Experiment 1						
Male	30·53	345·69	11·49	31·97	104·88	3·29
Female	26·38	331·8	12·61	27·16	86·69	3·19
Total	28·46	338·75	12·05	30·81	95·785	3·24
Experiment 2						
Male	35·15	376·79	10·79	33·88	115·44	3·45
Female	28·00	344·89	12·5	27·74	94·77	3·44
Total	31·58	360·84	11·645	30·78	105·1	3·45

### Experimental design

#### Experiment 1

Experiment 1 took place over 14 weeks. A total of forty Labrador retrievers were split into two dietary treatment groups, carnitine and control, with groups equalised between sex, body weight, genetics and body composition. The control group was supplemented with 4 g sugar and the carnitine group was supplemented with 3·75 g sugar and 250 mg Carniking™ brand l-carnitine powder. Dogs were fed a low-carnitine diet in which amounts were determined based on maintaining a minimum initial starting body weight. All feed offered and feed refusals were weighed to measure consumption. All dogs began a running exercise programme immediately after a baseline blood draw and body composition scan that included two short sprint runs ranging from 1·1 to 2·2 km and one long endurance run ranging from 8·8 to 16·1 km per week, until the final week where the dogs completed a final long endurance run of 24·2 km. All dogs wore global positioning system (GPS) and accelerometer collars during the running portion of the studies to quantify the distance ran and their activity output. Additional blood samples were collected from each dog before and 1  h after the final long run, and a final body composition scan was performed after the final long run. All dogs were weighed at the beginning of the study and weighed every 2 weeks throughout the study.

#### Experiment 2

Experiment 2 took place approximately 10 months after completion of experiment 1. Experiment 2 lasted 14 weeks and was slightly modified from experiment 1. A total of fifty-six Labrador retrievers were split into two dietary treatment groups that were equalised between sex, genetics and body composition. Of the dogs that were used in experiment 1, thirty-six went on to participate in experiment 2, and the treatment groups for those dogs were switched. The control group was supplemented with 4 g sugar and the carnitine group was supplemented with 3·75 g sugar and 250 mg Carniking™ brand l-carnitine powder. All dogs were fed a low-carnitine diet in which amounts were determined based on maintaining a minimum initial starting body weight. Feed offered and feed refusals were weighed to measure consumption. A baseline blood sample was collected, as well as the initial body composition scan, the day before beginning the running exercise programme. The exercise regimen included two long endurance runs per week ranging from 8·8 to 16·1 km, ending with a final long run of 24·2 km. Dogs wore accelerometer and GPS collars during all runs. All dogs were scanned after the final long run for body composition and had blood samples collected the day before the final long run, 1 h after the final long run, and 24 h after the final long run. All dogs were weighed at the beginning of the study and weighed every week throughout the study.

### Performance and running regimen

#### Experiment 1

All dogs completed a running programme for the duration of the experiment. Dogs wore an accelerometer collar (Actical^®^; Philips Respironics) to determine activity points per km (APKm) while running. All dogs ran short sprint runs twice per week, and a long endurance run once per week. The sprint running regimen was designed to simulate an American Kennel Club hunt test: the dogs ran multiple 100 m retrieves. The short sprints started at 1·1 km each session (multiples of 100 m retrieves), increasing incrementally for 10 weeks to 2·2 km and then tapering down until the final long endurance run in week 13. The long endurance runs started at 8·8 km, increasing incrementally for 10 weeks until dogs ran 16·1 km and then tapering down until the final 24·2 km long run. Dogs ran alongside an all-terrain vehicle and were free to run at their own pace, swim, play, etc. but met at least the minimum distance required.

#### Experiment 2

All dogs completed a running programme during the experiment. Dogs wore an accelerometer collar (Actical^®^; Philips Respironics) while running. For efficiency and for the prevention of heat-related injuries in the warm weather during experiment 2, retrieving sprints were not performed. Endurance running was preferable in hot weather as the dogs were able to swim and cool off at will. One of the runs each week included 100 m fartleks (periods of fast running intermixed with periods of slower running). The long runs started at 8·8 km, increasing incrementally for 10 weeks until 16·1 km and then tapering down until the final 24·2 km long run. Dogs ran alongside an all-terrain vehicle and were free to run, swim, play, etc. but met at least the minimum distance required.

### Body composition

All dogs were scanned using a GE Lunar dual-energy X-ray absorptiometry (DXA) (General Electric Company) machine for body composition before beginning each study to establish baseline, and after the final long run to view any changes. Dogs were anaesthetised for the scans using dexmedetomidine (Dexdormitor^®^; Zoetis Inc.), torbutrol (Zoetis Inc.) and atropine (Vedco Inc.). Dogs were positioned dorsoventrally for the scans and were closely monitored post-recovery.

### Blood collection and biomarker assays

All blood samples were collected from the dogs via the cephalic vein into EDTA vacutainers (Becton, Dickinson and Company). The baseline blood draw was performed before beginning any feeding, exercise, or supplementing regimen in a 24 h fasted state for both experiments. The pre-run blood draw was performed the day before the final long run, approximately 2 h after being fed 200 g of feed and designated supplements, for both experiments. The 1 h post-run blood draw was performed 1 h after the final long run, and approximately 3 h after being fed 200 g of feed and designated supplements, for both experiments. In experiment 2, a 24 h post-run blood draw was performed 24 h after the final long run and fed 200 g of feed and designated supplements approximately 2 h prior. All animals were monitored for signs of stress during blood collection procedures.

Blood samples were centrifuged according to assay kit instructions to collect blood serum. Serum was immediately frozen at −80°C until further use. Serum was evaluated for muscle protein excretion using CPK (Biovision Inc.) and myoglobin (Innovative Research Inc.) assay kits. Oxidative status was evaluated using TAC (Cayman Chemical Company) and TBARS (Cayman Chemical Company) assay kits. All samples were run in duplicate.

### Statistical analysis

GraphPad Prism 6.0 (GraphPad Software Inc.) was used to compare the effect of treatment groups on run time, food intake, body composition, body weight and changes in blood chemistry using an unpaired *t* test. JMP 10.0.2 (SAS Institute, Inc.) was used to create mixed, one-way and regression models for statistical analyses of the effect of treatment group on activity during endurance runs, experiment comparisons, food consumption aspects, body composition and blood chemistry. Experiment 1 and experiment 2 were analysed separately. Sex was analysed as a fixed effect based on the potential variance between male and female dogs. Results were considered significant if a *P* value of 0·05 or less was obtained.

## Results

### Feed intake

#### Experiment 1

Dogs in experiment 1 consumed an average of 651 g of feed per d. No significant difference was found overall between treatment groups (*P* = 0·1291; carnitine 626 (sem 24) *v*. control 677 (sem 23) g).

#### Experiment 2

Dogs in experiment 2 consumed an average of 536 g of feed per d. Carnitine dogs consumed significantly more weight of feed overall compared with the control group (*P* < 0·0001; 574 (sem 8·08) *v*. 540 (sem 8·08) g).

### Performance

#### Experiment 1

The carnitine group produced approximately 4000 more APKm overall during both the short sprint runs (*P* = 0·052) and the long runs (*P* = 0·0001) over 14 weeks compared with the control group (Table 4). The female carnitine group produced very intense activity overall at 56 354 APKm compared with the male carnitine group at 45 987 APKm, male control group at 46 099 APKm, and female control group at 47 780 APKm (*P* = 0·0009).

**Table 4.** Activity per km: experiment 1 (Mean values with their standard errors)

	Carnitine (*n* 20)		Control (*n* 20)		
	Mean	sem	Mean	sem	*P*
Sprints					
Female	51 844	1799	48 921·4	1342	0·1950
Male	52 978·3	1711	47 431·8	1313	0·0108
All	52 467·9	1239	48 101·4	941	0·0052
Long runs					
Female	56 433·4	1181	47 871·2	849	<0·0001
Male	45 953·4	750	45 996·2	1103	0·9745
All	50 827·8	753	46 843·9	717	0·0001

#### Experiment 2

No significant differences were found in APKm between the carnitine and control groups for all long endurance runs (*P* = 0·1754; 45 530 (sem 853) *v*. 47 344 (sem 1030) APKm) (Table 5).

**Table 5.** Activity per km: experiment 2 (Mean values with their standard errors)

	Carnitine (*n* 28)		Control (*n* 28)		
Long runs	Mean	sem	Mean	sem	*P*
Female	46 237	1098	47 172	1115	0·551199
Male	44 823	1307	47 543	1810	0·373029
All	45 530	853	47 344	1030	0·17536

### Body composition

#### Experiment 1

A significant increase in total body mass was observed from before the beginning of the study until the end for all dogs in both groups (*P* < 0·0001; 27·0 (sem 0·2502) *v*. 25·33 (sem 0·2502) kg). No significant differences in body composition were found between treatment groups (data not shown).

#### Experiment 2

Many more significant changes in body composition were noted in experiment 2. The carnitine group gained 0·74 kg total tissue mass while the control group lost 0·12 kg tissue mass (*P* = 0·0006). The carnitine group gained 0·68 kg lean mass (LM) while the control group lost 0·41 kg LM (*P* < 0·0001). From baseline to after the final long run, the female carnitine dogs had a change of 0·45 kg LM while the female control dogs lost 0·8 kg LM (*P* = 0·0006). Male carnitine dogs also gained 0·91 kg LM compared with only 0·09 kg gain in control males (*P* = 0·0050) (Table 6).

**Table 6.** Body composition: experiment 2 (Mean values with their standard errors)

	Carnitine (*n* 28)		Control (*n* 28)		
	Mean	sem	Mean	sem	*P*
Fat-initial (%)	15·58	1·2	16·02	1·35	0·8089
Male	14·31	1·16	15·19	1·53	0·6520
Female	16·85	2·1	16·74	2·17	0·9712
Fat-final (%)	16·62	1·06	17·5	1·23	0·5885
Male	14·81	1·05	14·9	1·75	0·9640
Female	18·43	1·75	19·75	1·54	0·5742
Fat-change (%)	1·04	0·71	1·48	0·73	0·6636
Male	0·49	0·61	−0·29	0·82	0·9712
Female	1·58	1·29	3·01	1·01	0·3893
Fat-initial (kg)	4·2	0·35	4·3	0·38	0·8533
Male	4·34	0·42	4·57	0·55	0·6520
Female	16·85	2·1	4·07	0·54	0·9997
Fat-final (kg)	4·59	0·33	4·62	0·35	0·9483
Male	4·56	0·36	4·49	0·62	0·9200
Female	4·62	0·57	4·74	0·39	0·8658
Fat-change (kg)	0·39	0·2	0·32	0·21	0·8207
Male	0·22	0·2	−0·08	0·28	0·3944
Female	0·56	0·35	0·67	0·27	0·7981
Lean mass-initial (kg)	22·56	0·74	22·23	0·69	0·7434
Male	25·63	0·58	24·68	0·75	0·3265
Female	19·59	0·69	20·1	0·76	0·5570
Lean mass-final (kg)	23·24	0·79	21·82	0·71	0·1879
Male	26·54	0·71	24·77	0·74	0·0973
Female	19·95	0·65	19·26	0·65	0·4648
Lean mass-change (kg)	0·68	0·16	−0·41	0·17	<0·0001
Male	0·91	0·22	0·09	0·15	0·0050
Female	0·45	0·21	−0·84	0·26	0·0006
Total mass-initial (kg)	26·76	0·83	26·53	0·78	0·8382
Male	29·97	0·75	29·25	1·07	0·5870
Female	23·56	0·86	24·17	0·71	0·5886
Total mass-final (kg)	27·5	0·84	26·41	0·79	0·3479
Male	30·67	0·72	29·25	1·05	0·2780
Female	24·34	0·94	23·95	0·7	0·7400
Total mass-change (kg)	0·74	0·16	−0·12	0·17	0·0006
Male	0·7	0·19	0·01	0·22	0·0268
Female	0·78	0·27	−0·22	0·25	0·0117
BMC-initial (g)	813·91	28·38	782·8	26·6	0·4273
Male	920·86	25·96	874·61	33·19	0·2824
Female	706·96	30·19	703·23	27·49	0·9278
BMC-final (g)	821·95	28·14	791·82	27·28	0·4455
Male	927·39	25·32	883·72	37·66	0·3447
Female	716·5	30·63	712·18	25·49	0·9145
BMC-change (g)	8·04	4·62	9·03	5·07	0·8859
Male	6·54	6·57	9·11	7·48	0·7981
Female	9·54	6·72	8·95	7·15	0·9531

BMC, bone mineral content.

### Recovery and oxidative status

#### Experiment 1

CPK assay results showed male carnitine dogs experienced a lower increase in enzyme secretion with a change of 5·54 mU/ml *v*. control male dogs at an increase of 12·94 mU/ml from before to after the final run (*P* = 0·05). Myoglobin assay results also showed that carnitine dogs had lower concentrations during the final long run (*P* = 0·0295; 6·69 (sem 2·7) *v*. 24·02 (sem 6·59) ng/ml) (Table 7).

**Table 7.** Change in biomarkers from pre- to post-run: experiment 1 (Mean values with their standard errors)

	Carnitine (*n* 20)		Control (*n* 20)		
	Mean	sem	Mean	sem	*P*
TAC (mm Trolox equivalents)	−0·03	0·01	−0·03	0·01	0·7709
Male	−0·03	0·01	−0·03	0·01	0·9765
Female	−0·04	0·01	−0·03	0·01	0·6496
TBARS (μm MDA)	0·41	0·71	2·14	0·96	0·1552
Male	1·3	0·9	1·7	0·4	0·7184
Female	−0·7	1·1	2·7	2·1	0·1727
Myoglobin (ng/ml)	6·69	2·7	24·02	6·59	0·0295
Male	7·04	3·9	13·91	6·74	0·4008
Female	6·11	3·51	37·91	11·34	0·0371
Creatine kinase (mU/ml)	9·3	1·86	13·64	2·28	0·1452
Male	5·54	2·63	12·94	2·4	0·0522
Female	13·06	2·06	15·58	6·03	0·6185

TAC, total antioxidant capacity; TBARS, thiobarbituric acid reactive substances; MDA, malondialdehyde.

#### Experiment 2

**Fig. 1.**
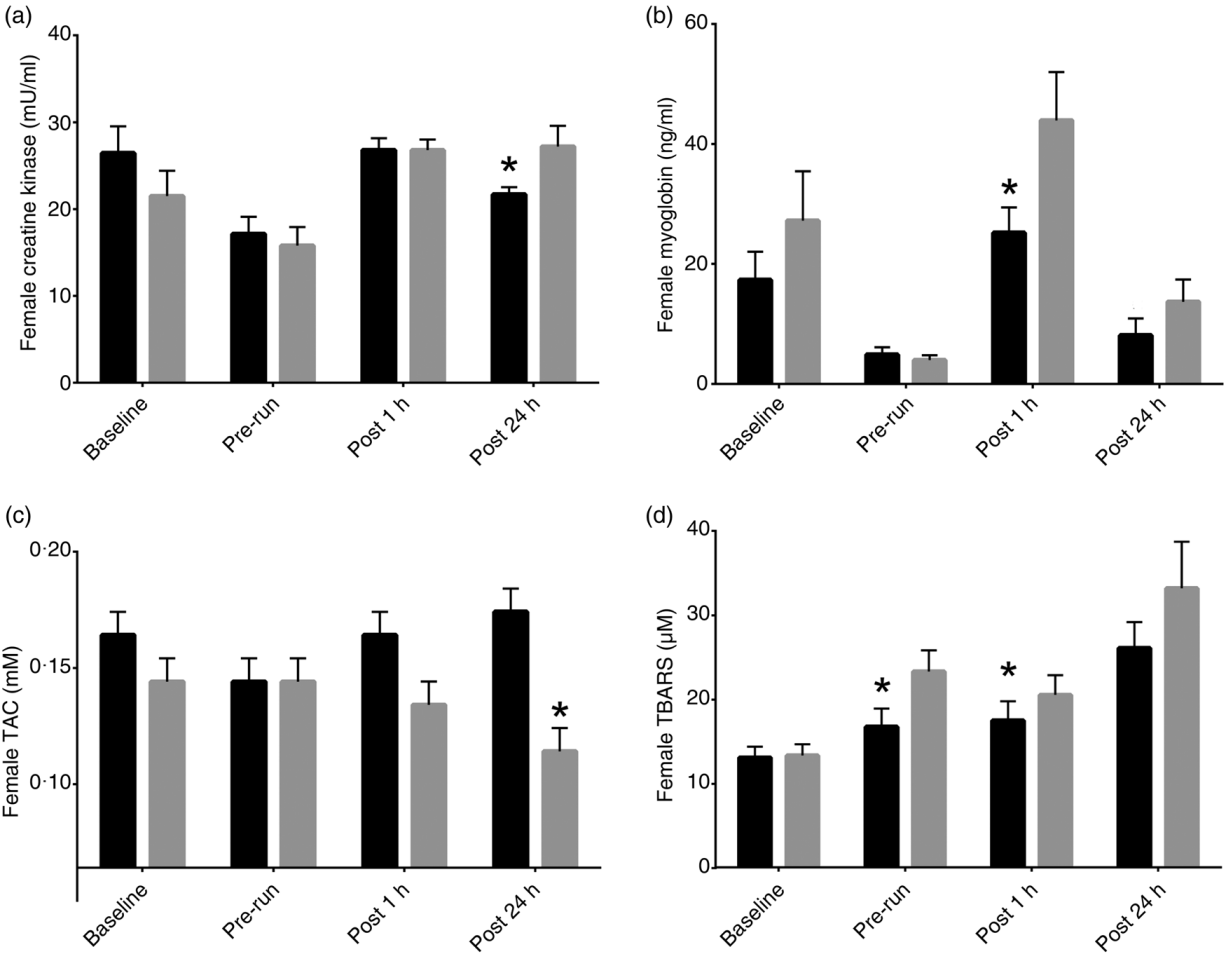
Female effect of l-carnitine in recovery biomarkers: (a) creatine kinase, (b) myoglobin, (c) total antioxidant capacity (TAC), (d) thiobarbituric acid reactive substances (TBARS). 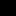, Carnitine; 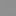, control. Values are means with standard errors represented by vertical bars. * Mean value was significantly different for carnitine compared with control (*P* < 0·05).

**Fig. 2.**
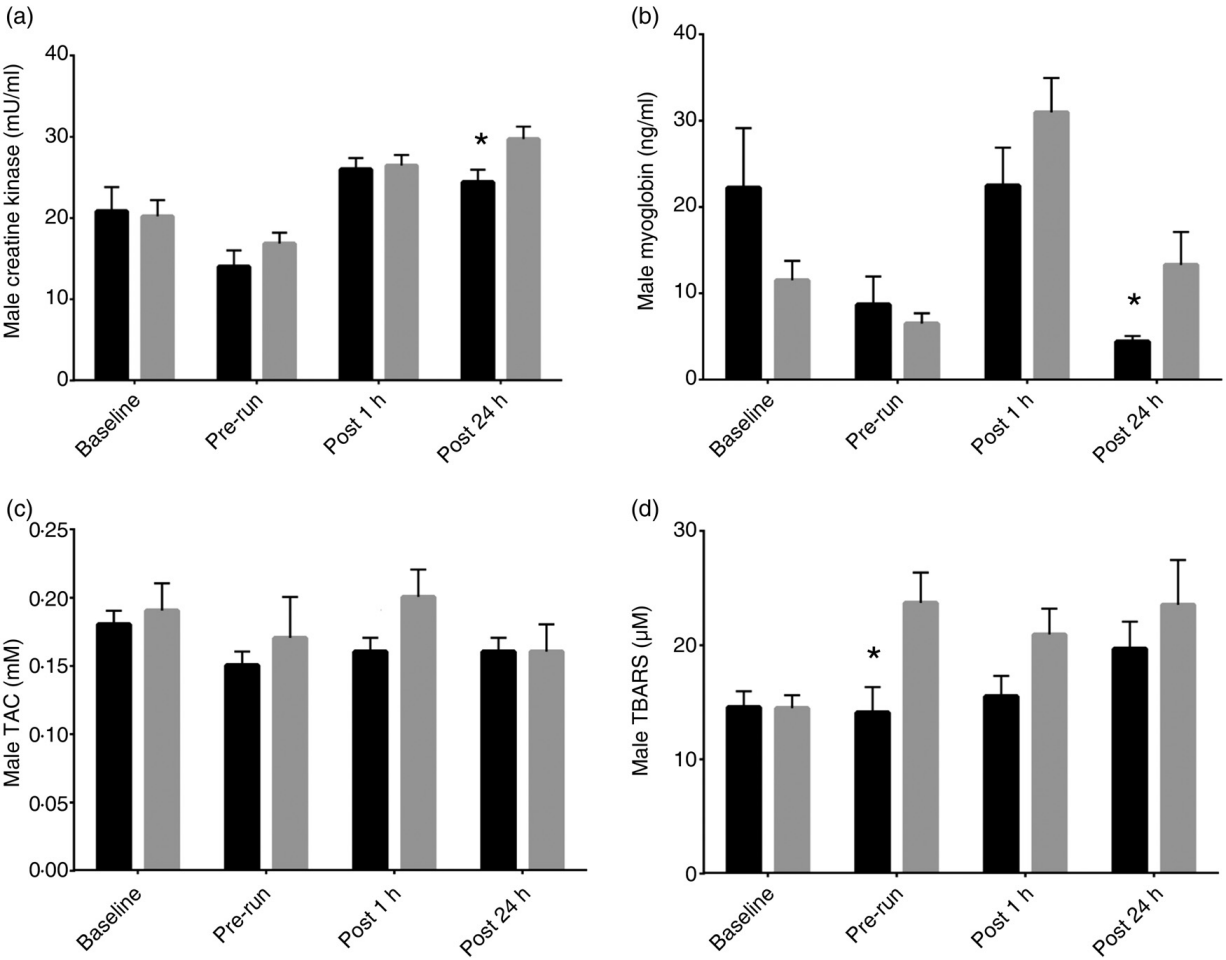
Male effect of l-carnitine in recovery biomarkers: (a) creatine kinase, (b) myoglobin, (c) total antioxidant capacity (TAC), (d) thiobarbituric acid reactive substances (TBARS). 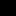, Carnitine; 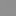, control. Values are means with standard errors represented by vertical bars. * Mean value was significantly different for carnitine compared with control (*P* < 0·05).

CPK results showed that carnitine dogs experienced significantly lower concentrations 24 h following the final long run at 23·06 mU/ml compared with the control group at 28·37 mU/ml (*P* = 0·003). Carnitine dogs had significantly lower myoglobin leakage compared with the control group both 1 h post-run (*P* = 0·0157; 23·83 (sem 3·02) *v*. 37·91 (sem 4·77) ng/ml) and 24 h post-run (*P* = 0·0189; 6·25 (sem 1·47) *v*.13·5 (sem 2·61) ng/ml) (Table 8). The female and male responses to l-carnitine both showed decreased levels of CPK and myoglobin compared with control. Female carnitine dogs had significantly lower myoglobin levels at 1 h post-run (*P* = 0·0491; 25·19 (sem 4·25) *v*. 43·94 (sem 8·03) ng/ml) while the males had a response at 24 h post-run (*P* = 0·0214; 24·4 (sem 1·53) *v*. 29·7 (sem 1·53) ng/ml) (Figs 1 and 2).

**Table 8.** Biomarkers in experiment 2 (Mean values with their standard errors)

	Carnitine (*n* 28)		Control (*n* 28)		
All dogs	Mean	sem	Mean	sem	*P*
Creatine kinase (mU/ml)					
Baseline	23·63	2·17	20·9	1·77	0·3356
Pre-run	15·58	1·4	16·28	1·28	0·7136
Post-run 1 h	26·39	0·96	26·63	0·88	0·8523
Post-run 24 h	23·06	0·88	28·37	1·45	0·0028
Myoglobin (ng/ml)					
Baseline	19·78	4·13	19·92	4·7	0·9819
Pre-run	6·78	1·74	5·13	0·73	0·3867
Post-run 1 h	23·83	3·02	37·91	4·77	0·0157
Post-run 24 h	6·25	1·47	13·5	2·61	0·0189
TBARS (μm)					
Baseline	13·77	0·94	13·81	0·87	0·9736
Pre-run	15·36	1·55	23·42	1·8	0·0013
Post-run 1 h	16·45	1·43	20·65	1·61	0·0561
Post-run 24 h	22·81	2·01	28·64	3·53	0·1568
TAC (mm)					
Baseline	0·17	0·01	0·16	0·01	0·6992
Pre-run	0·14	0·01	0·15	0·01	0·7755
Post-run 1 h	0·15	0·01	0·16	0·01	0·7495
Post-run 24 h	0·16	0·01	0·13	0·01	0·0496

TBARS, thiobarbituric acid reactive substances; TAC, total antioxidant capacity.

Carnitine dogs had significantly more TAC compared with control dogs 24 h post-run (*P* = 0·0496; 0·16 (sem 0·01) *v*. 0·13 (sem 0·01) mm) (Table 6). TBARS were significantly lower in carnitine dogs both before the final long run (*P* = 0·0013; 15·36 (sem 1·55) *v*. 23·42 (sem 1·8) µm) and 1 h after the long run (*P* = 0·056; 16·45 (sem 1·43) *v*. 20·65 (sem 1·61) µm) (Table 6). The females had a stronger response to l-carnitine. TAC levels were higher in carnitine dogs at 0·16 mm than control at 0·10 mm 24 h post-run (*P* = 0·0016). TBARS were significantly lower both pre-run (*P* = 0·0013; 16·68 (sem 2·16) *v*. 23·23 (sem 2·53) µm) and 1 h post-run (*P* = 0·0596; 17·44 (sem 2·26) *v*. 20·46 (sem 2·35) µm). Males did not have a positive response to l-carnitine in TAC levels, and TBARS levels were significantly lower in carnitine dogs only at the pre-run interval (*P* = 0·0104; 14·03 (sem 2·24) *v*. 23·64 (sem 2·66) µm) (Figs 1 and 2).

## Discussion

For the purpose of both experiments, APKm were obtained via accelerometers on each dog. Given that the dogs were free to run at their own pace for the prescribed distance, the activity points quantify the intensity of the exercise^(^^16^^)^. For instance, dogs frequently stopping or moving at a slow pace will have lower APKm *v*. dogs moving at a fast trot or run that will have higher APKm. Results from experiment 1 showed the carnitine dogs having a higher average APKm, but there were no significant outcomes for activity in experiment 2. Several factors could have affected this, such as the dogs in experiment 1 performing sprint running each week as well as the endurance exercise, or experiment 1 being performed during cooler months compared with experiment 2. Few other studies have been performed on l-carnitine's effect on exercise intensity; however, a study has been performed on the effect of l-carnitine on skeletal muscle force in canines. Dubelaar *et al.*^(^^17^^)^ found a 34 % increase in muscle force in the latissimus dorsi of dogs supplemented with l-carnitine. Alternatively, Trappe *et al.*^(^^9^^)^ compared l-carnitine's effects on velocity and time of high-intensity swimming in humans but did not find any significant effect on swimming time and velocity. This was attributed to the fact that the subjects used had been involved in swim training for at least 16 weeks and were at the capacity of their physiological limits. This may also be the case for the present study as the dogs were regularly exercised before beginning each experiment, and performed running exercise every week for the 14 weeks of each experiment.

Results of the body composition analysis in the present study showed no significant differences in experiment 1, but several significant differences in total tissue mass and LM in experiment 2. An explanation for the difference in results for each experiment may be due to the diet consumed during experiment 1 having higher intake than the diet consumed in experiment 2. Therefore, the dogs in experiment 1 did have a higher energy intake and should have had a reduced amount of food offered. The higher energy intake may have prevented l-carnitine's effects of decreasing fat and increasing LM. Experiment 1 was similar to a study in which human subjects underwent body composition scans before and after low-intensity cycling exercise during a 12-week period. Subjects in both l-carnitine and control groups were both overfed carbohydrates. The control group had an increase in total tissue mass solely due to an increase in total body fat, but the carnitine group did not have any increase in total tissue mass, total body fat or total LM^(^^18^^)^. In experiment 2 of the present study, the carnitine dogs consumed significantly more weight of food on average compared with the control dogs and were able to gain LM while the control dogs lost LM at the end of the running programme. The discrepancy between the present study and the Stephens *et al.*^(^^18^^)^ study may be the type of exercise performed, where the low-intensity cycling was not strenuous enough to induce a change in LM as observed in the present study 13-week running programme. The increased LM in the Labrador retrievers in experiment 2 may help prevent an increase in fat deposition, which is important for many life stages of dogs. Maintaining lean body mass in growing puppies can help prevent future obesity, as well as prevent the possible loss of LM as the dog ages^(^^19^^)^. l-Carnitine may prevent the loss of LM during increased activity and weight reduction, which is important for the long-term maintenance of optimum body condition^(^^20^^)^.

The findings in the present studies indicate a significant advantage for l-carnitine-supplemented dogs in preventing the loss of proteins that are indicative of muscle inflammation during strenuous exercise. Experiment 2's CPK assay results show that the carnitine group's CPK levels were significantly lower than the control group at 24 h post-run. The carnitine group's CPK levels were falling at this point while the control group's levels continued to rise. Parandak *et al.*^(^^21^^)^ examined human subjects’ performance during aerobic exercise via running, similar to the present study's exercise regimen with dogs, and found that l-carnitine supplementation resulted in lower CPK levels compared with control at the post-24 h exercise interval. Ho *et al.*^(^^22^^)^ also found both a significant reduction in CPK and myoglobin secretion in l-carnitine-supplemented subjects *v*. control during squat/leg press exercises. Volek *et al.*^(^^23^^)^ found a reduction in myoglobin release into the blood following squat-type exercise in subjects supplemented with l-carnitine. Volek *et al.*^(^^23^^)^ were unable to generate a CPK response to l-carnitine, although this was attributed to either a slow CPK response time or the inability to generate disruption to the sarcolemma due to type or intensity of exercise. Strenuous exercise causes disruption to the permeability of the sarcolemma, allowing proteins and enzymes such as CPK and myoglobin into the bloodstream. The time points at which a significant difference is noted between carnitine and control groups seems to vary slightly between species, sex and type of exercise performed but this evidence fully supports l-carnitine's benefit in exercise recovery. Future studies on the dose response in canines to supplemented l-carnitine may be necessary, as the effects seem to vary dependent on animal variables.

In experiment 2, the carnitine group experienced a steady increase in TAC values at each time interval when compared with the control group, which experienced a significant decrease in TAC at the post-24 h time interval. The present study shows that the control dogs had lost TAC well below baseline 24 h post-run while the carnitine dogs were able to maintain TAC at just under baseline. TBARS results were lower in carnitine dogs both before and after the final long run, indicating a much lower rate of lipid peroxidation taking place not only during the oxidative stress of the final long run, but during normal daily activity as well. Females had an overall stronger response to l-carnitine and a significantly lower average body weight, indicating that a higher dosage of l-carnitine may be necessary to aid the males’ oxidative status.

Parandak *et al.*^(^^21^^)^ examined l-carnitine's effects on oxidative stress via TAC and TBARS in human subjects after running exercise and found similar results; TAC was higher and TBARS were lower in l-carnitine-supplemented subjects 24 h post-run. During strenuous exercise, reactive oxygen species (free radicals) are produced due to increased oxygen consumption and anaerobic conditions in the muscle tissue^(^^24^^)^. l-Carnitine supports more efficient use of oxygen and energy in muscle tissue, thereby minimising the loss of oxygen and keeping the tissue more aerobic^(^^25^^)^.

### Conclusion

Supplementation of l-carnitine had positive impacts on the performance and recovery of Labrador retrievers in both experiments. Findings from the studies performed indicated that l-carnitine has beneficial effects on LM and intensity (activity) of exercise. These effects seem to be more pronounced in females than in male dogs, possibly because of different requirements in the dosage. l-Carnitine also prevented exercise-induced muscle damage based on the reduced efflux of inflammatory enzymes and reduced oxidative stress during strenuous exercise in Labrador retrievers.
